# MOTS-c: A promising mitochondrial-derived peptide for therapeutic exploitation

**DOI:** 10.3389/fendo.2023.1120533

**Published:** 2023-01-25

**Authors:** Yuejun Zheng, Zilin Wei, Tianhui Wang

**Affiliations:** ^1^ Environmental and Operational Medicine Research Department, Academy of Military Medical Sciences, Academy of Military Sciences, Tianjin, China; ^2^ Tianjin Key Lab of Exercise Physiology and Sports Medicine, Tianjin University of Sport, Tianjin, China

**Keywords:** MOTS-c, mitochondrial-derived peptide, therapeutic exploitation, synthetic biology, endocrine

## Abstract

Mitochondrial ORF of the 12S rRNA Type-C (MOTS-c) is a mitochondrial-derived peptide composed of 16 amino acids encoded by the 12S rRNA region of the mitochondrial genome. The MOTS-c protein is transferred to the nucleus during metabolic stress and directs the expression of nuclear genes to promote cell balance. Different tissues co-expressed the protein with mitochondria, and plasma also contained the protein, but its level decreased with age. In addition, MOTS-c has been shown to improve glucose metabolism in skeletal muscle, which indicates its benefits for diseases such as diabetes, obesity, and aging. Nevertheless, MOTS-c has been used less frequently in disease treatment, and no effective method of applying MOTS-c in the clinic has been developed. Throughout this paper, we discussed the discovery and physiological function of mitochondrial-derived polypeptide MOTS-c, and the application of MOTS-c in the treatment of various diseases, such as aging, cardiovascular disease, insulin resistance, and inflammation. To provide additional ideas for future research and development, we tapped into the molecular mechanisms and therapeutic potentials of MOTS-c to improve diseases and combined the technology with synthetic biology in order to offer a new approach to its development and application.

## Introduction

Mitochondria are organelles produced by archaea that are required for the production of ATP (1). The organism exhibits semi-autonomous genetic systems, independent genomes, and unique genetic codes that are similar to those found in bacteria (2). Recently, a short open reading frame (sORF) encoded in the mitochondrial genome has been discovered (3, 4). These sORF produce bioactive peptides, collectively known as mitochondrial-derived peptides (MDP), which have a wide range of physiological functions and can explain how mitochondria communicate within and between cells in a specific disease environment (5). Mitochondrial-derived peptides may answer the key biological problems that have plagued the field for decades (such as mitochondrial-nuclear communication, metabolic dysfunction, etc.) (6). Whether in the form of mitochondrial-derived peptide itself or in terms of sORF, mitochondrial-derived peptide is suitable for research as a therapeutic agent (1).

Studies discovered mitochondrial-derived peptide called MOTS-c has been shown to significantly reduce the level of pro-inflammatory factors in mice and increase anti-inflammatory factors and insulin-stimulated glucose treatment rates, as well as glucose homeostasis (7–9). Furthermore, human studies showed that exercise increased MOTS-c levels in skeletal muscle and blood circulation, indicating that MOTS-c is a mitochondrial-derived peptide induced by skeletal muscle exercise (9, 10). Additionally, more and more studies have revealed the importance of MOTS-c in regulating obesity and diabetes (11, 12), longevity (13), and cardiovascular disease (14). Specifically, this paper discusses the application of mitochondrial-derived peptides, including MOTS-c, in the treatment of diseases and anticipates the future development direction of MOTS-c combining synthetic biology to provide new ideas on how it can be developed and applied.

## Physiological function of MOTS-c

**Figure 1:**
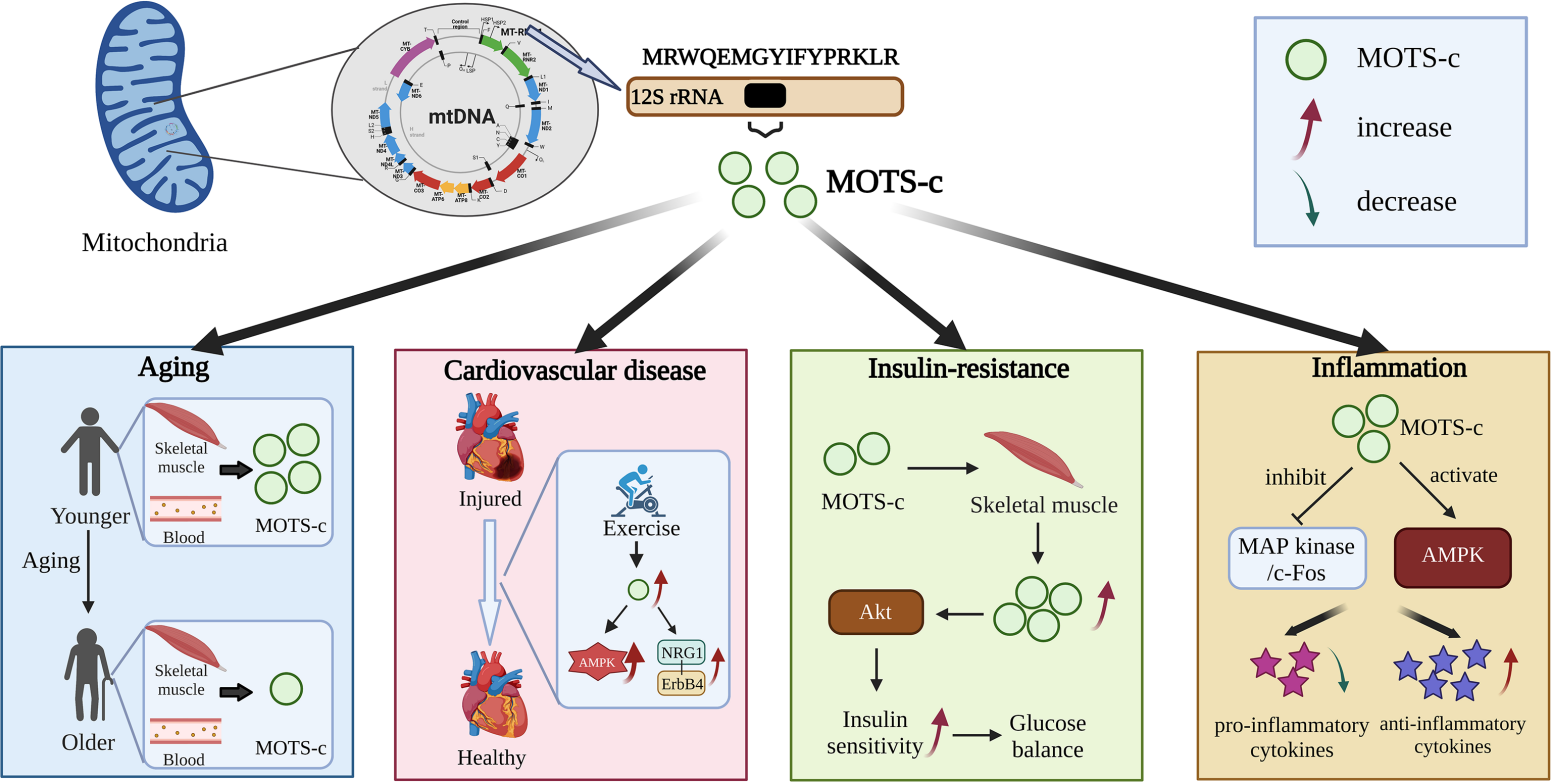
Effects of MOTS-c against diseases.

MOTS-c, one of the newly discovered sORF-encoded peptides, is a 16-amino acid polypeptide encoded by the mitochondrial 12S rRNA gene and localized to mitochondria under resting conditions (**Figure 1**) (7). Translation of MOTS-c peptide occurs exclusively in the cytoplasm, as mitochondrial translation, using the mitochondria-specific genetic code, results in tandem codons. As a result, the polyadenylated transcript would be exported from the mitochondria. The sequence of MOTS-c peptides, especially the first 11 residues, is highly conserved among 14 species, including humans and mice (7).

MOTS-c, as a mitochondrial coding regulator, has endocrine-like and nuclear transcriptional regulation on muscle metabolism (10, 15), insulin sensitivity (16, 17) and body weight (18). Approximately 11.9-fold increase in endogenous MOTS-c levels have been found in skeletal muscle following exercise (compared to pre-exercise values), and this increase can remain for 4 hours following exercise (19). In addition, circulating endogenous MOTS-c levels increase 1.6-fold during exercise, 1.5-fold after exercise, and then return to baseline levels after 4 hours. These results imply that exercise stimulates the expression of MOTS-c, which is encoded by mitochondria, in humans (19). Exercise is accomplished through a signal network that crosses physiological functions in real time. Those with limited movement can benefit tremendously from kinesimatology, which also accelerates metabolism (10). MOTS-c, as a new type of mitochondrial signal molecule, may stimulate exercise-mediated physiological responses to increase endurance (13). Therefore, MOTS-c can be used as a motion simulator to mediate the function of the motion signal system (20).

Originally, MOTS-c was identified in the process of genetic and pharmacological screening of metabolic regulation in human cells (21). In fact, its metabolic effects under various pathophysiological conditions have been confirmed by several studies. It has been reported that MOTS-c can promote the entry of glucose into cells through 5’-monophosphate-activated protein kinase (AMPK) pathway to participate in glycolysis (22). Moreover, MOTS-c can improve insulin sensitivity of skeletal muscle and inhibit weight gain and insulin resistance caused by high-fat diet (6). However, human experiments confirmed that only obese Chinese male children/adolescents (5-14 years old) had significantly lower (20.3%) intra-cycle MOTS-C levels (22). In a cohort study, plasma MOTS-c levels in men were negatively correlated with fasting insulin levels, glycosylated hemoglobin and body mass index (23). However, another cohort study of 31- to 38-year-old adults found no correlation between body mass index and plasma MOTS-c levels (10). This contradiction may be related to the individual differences of the subjects.

MOTS-c has also been found to be involved in the regulation of nuclear gene expression by binding to transcription factors (5, 19, 24). Under resting conditions, MOTS-c is mainly distributed in the mitochondria outside the nucleus (5, 7, 19). When metabolic stress occurs, MOTS-c in cells can be transferred to the nucleus in an AMPK-dependent manner and bind to transcription factors regulated by ARE, thus improving the stress resistance of cells (16, 24). In addition, the entry of MOTS-c into the nucleus requires hydrophobic groups, which means that MOTS-c may need the help of other proteins that need further verification (5, 25). Furthermore, MOTS-c plays the role of endocrine-like factors by regulating nuclear transcription to restore homeostasis. The function may have important implications for age-related diseases in response to metabolic stress by promoting intracellular homeostasis, and MOTS-c gene polymorphisms have been found to be associated with human lifespan (18, 20). Moreover, MOTS-c may improve diabetes by inhibiting insulin resistance and diet-induced obesity (26). And MOTS-c can promote glucose utilization, inhibit oxidative stress, activate NF-κB to inhibit inflammation, and effectively protect coronary artery endothelial cell dysfunction (9).

## Effects of MOTS-c against diseases

### MOTS-c and aging

Gradually disordered metabolic level is one of the signs of aging, which inhibit the normal physiological function of the body and even lose the ability to take care of themselves (27). The reality is that aging is a key risk factor for chronic diseases (28). Adaptation of cellular responses to changing internal and external environments is necessary for the health of an organism. Besides generating large amounts of cellular energy, mitochondria are closely related to aging, but the mechanism behind this phenomenon is unclear.

Studies have shown that the interaction of MOTS-c/NRF2 can improve the expression of mitochondrial protective genes (5, 28, 29). The aging process could lead to a decrease in MOTS-c levels (28, 29). In fact, MOTS-c levels in skeletal muscle and blood circulation in both humans and mice decrease with age. Studies have shown that blood MOTS-c levels in young people are 11% and 21% higher than those in middle- and old-aged people, respectively (30). In addition, different from animal experiments, the levels of MOTS-c in skeletal muscle of the elderly were the highest, indicating that the level of MOTS-c in plasma and muscle decreased gradually with age (30). This phenomenon may be attributed to the differential regulation of tissue specificity. Furthermore, the strong correlation between pathological results of different ages and the level of MOTS-c suggests that higher MOTS-c is beneficial to delaying aging.

### MOTS-c and cardiovascular disease

Obesity is the main culprit of cardiovascular problems (31). Clinical evidence showed that nearly 1/3 of severely obese people suffered from heart failure. And with the extension of the duration, the prevalence rate will gradually increase, and the prevalence rate will exceed 90% after 30 years (16, 32). Furthermore, obesity would lead to ventricular remodeling and dysfunction, which destroy the structure and physiological function of the heart, and eventually lead to heart failure (32). A growing body of research has shown a close relationship between MDP and the above factors, and MDP improved the pathological response of cardiovascular disease (CVD) through a variety of mechanisms (18, 24).

Recent studies have shown a protective effect of MOTS-c against cardiac dysfunction and pathological remodeling (33). Peng Zhong reported that MOTS-c prevented the development of heart failure *via* the activation of the AMPK pathway (34). Furthermore, Ismail Laher revealed that both aerobic exercise and MOTS-c can improve heart structure and function, thereby protecting the health of cardiovascular (33). Functional enrichment analysis showed that MOTS-c improved angiogenesis, inflammation and apoptosis in terms of cell function, suggesting that MOTS-c may have the same effect as aerobic exercise and improve heart failure in patients with diabetes through NRG1-ErbB4 pathway (14). This study reveals a new pathway for MOTS-c to protect against cardiovascular disease. It has also been observed that the addition of exogenous MOTS-c increases the level of myocardial MOTS-c, which activates AMPK (33). In cardiomyocytes, however, the target of MOTS-c is not clearly known, and more studies are needed to uncover its function.

### MOTS-c and insulin-resistance

Insulin resistance can lead to a decrease in the number and the abnormal morphology of mitochondria in tissue cells, which in turn hinders the synthesis of ATP (18). MOTS-c is described as a “motion simulator” that systematically regulates glucose metabolism in the body and the role of muscle insulin (15).

MOTS-c enhances insulin sensitivity throughout the body through muscles (24). Previous studies have revealed that MOTS-c can enhance the insulin sensitivity of skeletal muscle and improve the utilization of glucose (16, 24). The addition of MOTS-c activated the Akt pathway in mouse skeletal muscle, which further positively regulated the expression of MOTS-c. Given that 70% of Mel and 85% of insulin-stimulated glucose disposal enters the skeletal muscle, the enhancement of insulin sensitivity and glucose balance by MOTS-c may be mediated in this tissue. In addition, aging leads to increased insulin resistance, which reduces MOTS-c levels in skeletal muscle and blood of mice (15). It has been reported that MOTS-c improves age-related insulin resistance in male mice by increasing glucose intake in soleus muscles (20). The results showed that the muscles of old mice were more resistant to insulin than those of young mice. Interestingly, the insulin sensitivity of old mice was the same as that of young mice after 7 days of MOTS-c intervention (7). In light of MOTS-c’s role in increasing insulin sensitivity and glucose balance, some studies have tested the effects of MOTS-c on inbred CD-1 mice fed a high-fat diet (HFD). The results showed that MOTS-c treatment prevented obesity in mice fed a high-fat diet, but did not affect the weight of mice fed a normal diet (7). In addition, MOTS-c can improve blood glucose balance and prevent hyperinsulinemia caused by high-fat diet. A major benefit of MOTS-c is that it affects muscle tissue directly, making it the most effective treatment for insulin resistance. A major advantage of MOTS-c is that it can avoid hepatotoxicity associated with metformin, AICAR, or methotrexate, which means it is a potential therapeutic target (24).

### MOTS-c and inflammation

By analyzing the changes in inflammatory cytokines in mice’s serum, the analgesic effect of MOTS-c licking time was evaluated, as well as its anti-inflammatory effects (8). The results demonstrated that intraperitoneal injection of MOTS-c could reduce the licking time in the second phase of the formalin test in a dose-dependent manner. Compound C, an AMPK antagonist, weakened the analgesic effect of MOTS-c (9, 35). A significant decrease in pro-inflammatory cytokines and an increase in anti-inflammatory cytokines was observed with MOTS-c in mice serum (8). In addition, MOTS-c treatment significantly increased the phosphorylation level of AMPK α and inhibited the activation of extracellular signal-regulated kinase (ERK), c-Jun N-terminal kinase (JNK), P38, and c-Fos expression induced by formalin (8). These results suggested that the analgesic and anti-inflammatory effects of MOTS-c were through activation of AMPK pathway and inhibition of MAP kinase/c-Fos pathway. MOTS-c, as a small molecular active peptide, has been reported to have potential applications in aging, insulin resistance, cardiovascular disease, and inflammation. In the future, the use of synthetic biology technology to introduce MOTS-c into probiotics to achieve its accurate and controllable expression is of great significance to human health and the prevention of various diseases.

## Development and application of MOTS-c

With the rapid development of synthetic biology technology, the role of genetic engineering bacteria in the treatment of various diseases has become more and more prominent (36, 37). Genetically engineered bacteria are bacteria that use DNA recombination technology to transfer the target gene into bacteria (such as *E. coli*) to express and produce the desired protein (38, 39). Engineered bacteria can achieve targeted gene reprogramming, selective functional recombination and precise space-time control, so they are widely used in medical and pharmaceutical industries (40). Genetically engineered bacteria have been developed as diagnostic and therapeutic tools for the treatment of many diseases, including cancer (41), diabetes (38), inflammatory bowel disease (42, 43) and viral infection (44). Chen Zhiyi of Nanhua University and others designed a kind of ultrasound-responsive bacteria, which can induce the expression of foreign gene IFN-γ in an ultrasound-controlled way, and improve the anti-tumor efficacy of the engineering bacteria *in vitro* and *in vivo* (42). AmirZarrinpar of the University of California genetically engineered natural *E. coli* isolated from the intestines of mice to express specific genes and improve diabetes (38). In addition, the use of bacteria as carriers to deliver drugs to tumors and other lesions, or to modify bacteria to express or deliver targeted drugs will greatly improve the therapeutic effect (39, 45, 46). In addition to using genetically engineered bacteria as drug delivery carriers, extracellular vesicles (EVs), especially exocrine bodies, originate from cells through exocytosis and are absorbed by target cells, which can transmit biological signals or even deliver drugs between local or distant cells (47). However, accurate, efficient and selective identification, separation and quantification of exocrine remains a challenge (48). Compared with exosomes, genetically engineered bacteria have stronger biological activity, higher effective drug concentration and more stable drug structure in drug delivery.

**Figure 2:**
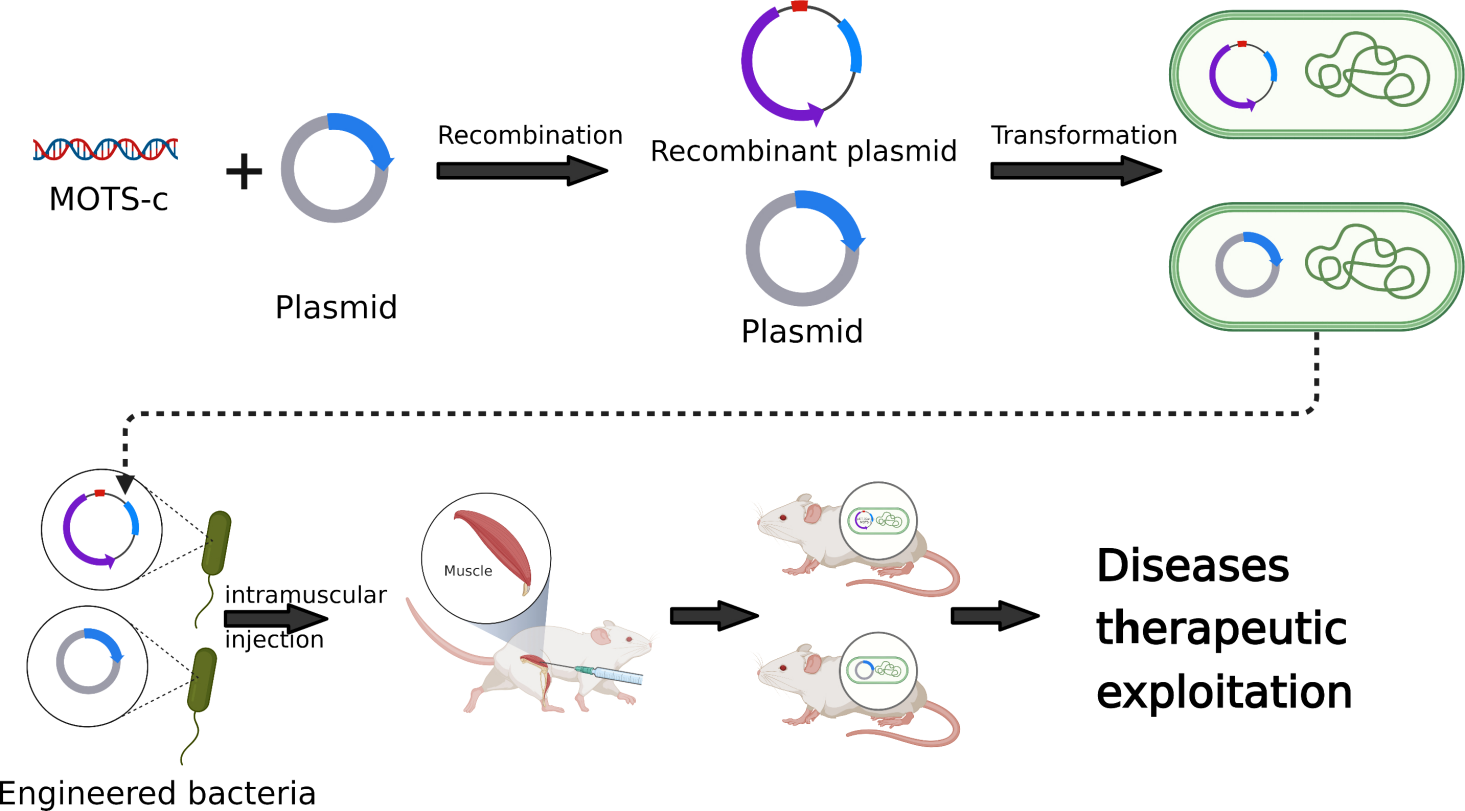
Development and application of MOTS-c in synthetic biology.

MOTS-c, as a small molecular active peptide, has been reported to have potential applications in aging, insulin resistance, cardiovascular disease and inflammation (**Figure 1**). In the future, the use of synthetic biology technology to introduce MOTS-c into probiotics to achieve its accurate and controllable expression is of great significance to human health and the prevention of various diseases (**Figure 2**). However, the virulence and uncontrollable immune response of bacteria greatly limit the clinical trial and application of bacterial therapy. It is an urgent problem for researchers to improve the safety and therapeutic effect of bacteria (37). At present, the most commonly used methods are gene-modified bacteria and surface-modified bacteria, and some natural bacteria such as *Lactobacillus* (LAB) and *Escherichia coli* Nissle1917 (EcN) had been proved to be safe clinically (49, 50). Using these bacteria as chassis bacteria will greatly improve the clinical effectiveness.

## Conclusion

Recently, the positive effects of MOTS-c, one of the mitochondria-derived peptides, on various diseases have been gradually discovered and reported. MOTS-c targets skeletal muscle and can enhance glucose metabolism. Therefore, MOTS-c plays an important role in the regulation of cardiovascular, diabetes, exercise and longevity. It is a new mitochondrial signaling mechanism and plays a role in regulating intracellular and intercellular metabolism. It has been reported that MOTS-c is a kind of exercise-induced mitochondrial coding regulator, and the level of MOTS-c in skeletal muscle and blood of mice decreases with age. Systemic injection of MOTS-c can restore the level of MOTS-c in aged mice and successfully reverse age-related skeletal muscle insulin resistance. At the same time, muscle cells overexpressing MOTS-c can improve glucose uptake, which is mainly related to the activation of AMPK pathway. Additionally, as an endocrine factor, MOTS-c is likely to exert its effects through cellular entry, which is an ongoing investigation at multiple ends, including its cellular uptake. Understanding the detailed molecular details of MOTS-c is an ongoing endeavor. It was reported that after 30 minutes of treatment, MOTS-c can exert its effects by entering the cells and exerting its effects. There is still a great deal of unclarity as to how MOTS-c enters cells without being degraded and retains its biological activity. A future study of the mechanism by which MOTS-c enters cells will be of great importance in order to determine how it exerts its clinical therapeutic effect. As a potential target for treatment development, MOTS-c is expected to be used in the treatment development of a variety of diseases. Synthetic biology techniques, such as gene editing and genetic engineering, can greatly improve biological activity and deliver MOTS-c directly to the acting site, thus further expanding the therapeutic application of MOTS-c. However, the subsequent biosafety problem has always been the focus of attention. How to reduce or eliminate the toxicity of genetically engineered bacteria to the body is a key scientific problem to be solved urgently.

## Author contributions

YZ: writing—original draft. ZW: review and editing, supervision. TW: review and editing, supervision. All authors have read and agreed to the published version of the manuscript. All authors contributed to the article and approved the submitted version.
